# Traumatic injuries of thigh and calf muscles in athletes: role and clinical relevance of MR imaging and ultrasound

**DOI:** 10.1007/s13244-012-0190-z

**Published:** 2012-09-16

**Authors:** Daichi Hayashi, Bruce Hamilton, Ali Guermazi, Richard de Villiers, Michel D. Crema, Frank W. Roemer

**Affiliations:** 1 Department of Radiology, Boston University School of Medicine, 820 Harrison Avenue, FGH Building, 3rd Floor, Boston, MA 02118 USA; 2 Department of Radiology, Klinikum Augsburg, Augsburg, Germany; 3 Department of Radiology, Hospital do Coração (HCor) and Teleimagem, São Paulo, SP Brazil; 4 ASPETAR, Qatar Orthopaedic and Sports Medicine Hospital, Doha, Qatar; 5 Van Wageningen & Partners, Somerset West, South Africa

**Keywords:** Muscle injury, Thigh, Calf, MRI, Ultrasound

## Abstract

**Objectives:**

Magnetic resonance (MR) imaging and ultrasound have become valuable tools for evaluation of traumatic muscle injuries in athletes. Common athletic injuries include strain, contusion and avulsion, which are characterised by muscle fibre disruption, intramuscular haemorrhagic dissection, haematoma at the musculotendinous junction, and perifascial blood or haematoma.

**Methods:**

MR imaging may allow clinicians to predict the time required before athletes can return to competition and the risk of injury recurrence.

**Results:**

Fluid-sensitive MR sequences, e.g., fat-suppressed T2-weighted or proton density-weighted turbo spin echo (TSE), and short-tau inversion recovery (STIR) sequences are suitable for detecting oedematous changes in the musculotendinous unit, and for delineating intramuscular or perifascial fluid collections or haematoma. T1-weighted spin echo sequences are used to visualise atrophy and fatty infiltration and to differentiate between haemorrhage/haematoma and oedema. While ultrasound may play a role as an adjunctive imaging method, it is less accurate than MR images for assessing the extent of the injury and it cannot differentiate between new and old injuries.

**Conclusions:**

In this pictorial review, imaging features of lower extremity muscle injuries including strain, contusion and avulsion are reviewed, focusing on MR and ultrasound imaging findings after initial injury and during follow-up, and their relevance in clinical practice is discussed.

**Teaching points:**

• *MR imaging may allow clinicians to predict time required before athletes can return to competition*

• *Fluid-sensitive MR sequences are suitable for detecting oedematous changes in the muscles*

• *T1-weighted sequences are used to differentiate between haemorrhage/haematoma and oedema.*

• *Ultrasound can also be used but is less accurate than MR imaging for assessing the extent of the injury*

## Introduction

Magnetic resonance (MR) imaging has been applied to muscle injuries for more than a decade [1–4]. With the development of more sophisticated scanners and imaging protocols [5], MR imaging has become a valuable tool for evaluation of traumatic muscle injuries. Football players, dancers, track and field, and other competitive athletes are at a high risk for acute muscle injury of the lower extremities due to high-speed running and stretching to extreme joint positions during such sport activities [6]. Although clinical examinations remain very important, it has been shown that radiological findings can aid clinicians in both the initial assessment and follow-up [7]. Appropriate management decisions, return to training and competitions, and prediction of injury recurrence may all be enhanced with appropriate imaging [8–12]. Ultrasound remains a popular alternative imaging modality for the assessment of muscle injury [13] and has some advantages over MR, i.e., lower cost and greater availability, short imaging time, no contraindications (e.g., pacemakers), and capability for dynamic imaging and comparison with the contralateral side. However, overall its limitations, including user dependence and lower sensitivity [13], tend to outweigh its advantages as far as accurate pathology delineation in elite athletes is concerned.

Muscle injuries are responsible for a large proportion of time lost to competition [14], and for all professional athletes rapid return to training and competition is a priority. However, it is also important not to return to competition too soon, when the risk of recurrent injury is still very high [15]. Clinicians and sports medicine physicians play a key role in establishing both an accurate prognosis and a return to training and competition pathway, and both MR imaging and ultrasound are useful adjuncts to the clinical assessment.

In this review article, we will briefly describe the anatomy of the lower extremity muscles at risk for injury according to their groups (i.e., hamstring muscles, adductor muscles, quadriceps muscles, and calf muscles). Secondly, we will discuss different types of musculotendinous injuries (i.e., musculotendinous strain, muscle contusion, and avulsion injury). Thirdly, characteristics of both MR imaging and ultrasound will be described, including advantages and limitations of each. Fourthly, we will illustrate each type of muscle injury described earlier with examples of MR and ultrasound images. Finally, the natural course of muscle injuries treated non-surgically will be illustrated with examples.

## Muscles of the lower extremities

### Hamstring muscle group

This group of muscles occupies the posterior compartment of the thigh and consists of the long and the short heads of the biceps femoris laterally and the semimembranosus and the semitendinosus muscles medially. Hamstrings cross both hip and knee joints, and integrate extension at the hip with flexion at the knee. The greatest musculotendinous stretch is incurred by the biceps femoris [16], which may contribute to its tendency to be the most commonly injured muscle in the hamstring muscle group, especially during high-speed running [17]. The semitendinosus muscle is a thin, band-like muscle with a long tendon distally, which may predispose the muscle to rupture [18]. The semimembranosus muscle arises from the superolateral part of the ischial tuberosity. It is important not to mistake this muscle for the semitendinosus muscle, since the proximal tendon of the latter may not always form a distinct structure.

### Adductor muscles

Hip adductors comprise, from lateral to medial, the pectineus, the adductors (longus, brevis, and magnus) and the gracilis. Of this group, the adductor longus is the most commonly injured [19]. This muscle is thought to be most susceptible to overstretching during such movements as lunging for a ball or sidestepping an opponent during a football or a rugby match. Injury may also be caused by sudden resistance to a strong adduction force, such as an opponent blocking forceful hip adduction in a block tackle [19].

### Quadriceps muscles

The quadriceps muscles occupy the anterior compartment of the thigh and comprise the rectus femoris, vastus medialis, vastus lateralis and vastus intermedius. Proximally, the straight head of the rectus femoris originates from the anterior inferior iliac spine, while the reflected head originates from the groove just above the acetabulum. Distally, these four muscles converge to insert on the superior pole of the patella as the trilaminar quadriceps tendon [20]. Of the quadriceps muscles, the rectus femoris muscle is the most commonly injured [9, 21]. This is thought to be because it crosses both the hip and knee joints, contains a high percentage of type II fibres, and has a complex musculotendinous architecture [22–24].

### Calf muscles

The calf muscle complex occupies the posterior compartment of the distal lower limb and includes the medial and the lateral heads of the gastrocnemius, and the soleus, which are collectively known as the triceps surae. The two heads (medial and lateral) of the gastrocnemius unite into a broad aponeurosis, which eventually unites with the deep tendon of the soleus to form the Achilles tendon [25]. The gastrocnemius is considered at high risk for injury because it crosses the knee and ankle joints and has a high density of type II fibres [21, 26]. The soleus is considered low risk for injury because it only crosses the ankle joint and is largely comprised of type I fibres [27]. The plantaris muscle crosses the knee and ankle joints before it joins the Achilles tendon insertion on the posterior surface of calcaneus. However, the plantaris is thought to be largely vestigial and is rarely involved in calf injuries [26, 27], and thus is not included in this review.

### Modalities for imaging of musculotendinous injuries

#### Magnetic resonance imaging

**Fig 1:**
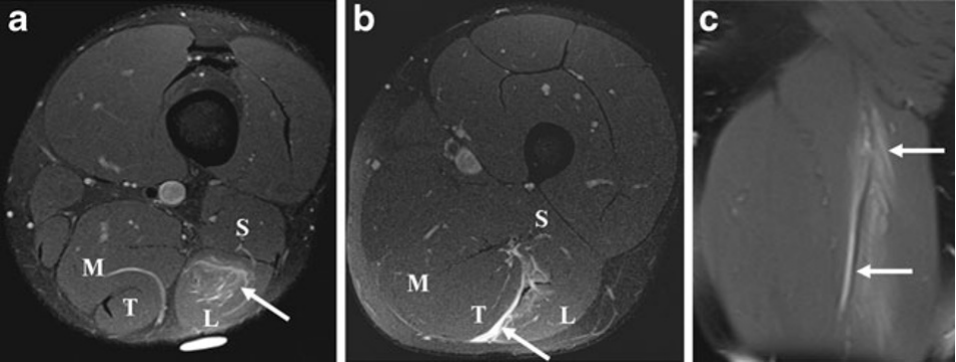
A 21-year-old sprinter presenting with a left hamstring injury, clinically diagnosed as a grade 1 injury. **a** Axial FS PD-w TSE image shows an area of hyperintensity with a feathery appearance (*arrow*), consistent with a strain of the long head of the biceps femoris (*L*). A marker is placed to indicate the location of tenderness. **b** Epifascial oedema (*arrow*) is noted between the long head of the biceps femoris (*L*) and the semitendinosus (*T*). **c** Coronal FS PD-w TSE image demonstrates the extent of injury in the longitudinal direction (*arrows*)

Following musculotendinous injuries, MR images are commonly acquired to locate the lesion and assess its severity. MR, together with ultrasound, is currently considered the modality of choice in the assessment of musculotendinous injuries [7]. Under normal circumstances, images from only the affected leg are acquired using a surface coil, but the appropriate coil should be selected to obtain the desired field of view. Imaging of the contralateral leg is performed in exceptional cases only (e.g., bilateral injury). Contrast enhancement is rarely needed except to distinguish solid from cystic lesions or to diagnose muscle infarction [2]. To correlate imaging with clinical findings, a skin marker (e.g., a capsule filled with fish oil or vegetable oil, Fig. 1a) is placed over the area of symptoms. Extent of injuries and associated architectural distortion is assessed using axial, sagittal and coronal images oriented along the long and short axes of the involved musculotendinous unit. The axial plane is useful to assess muscle contours and to delineate the musculotendinous junction and its exact anatomical relation with focal lesions [2], while coronal and sagittal planes are used to assess the longitudinal extent of injury [3].

**Fig 2:**
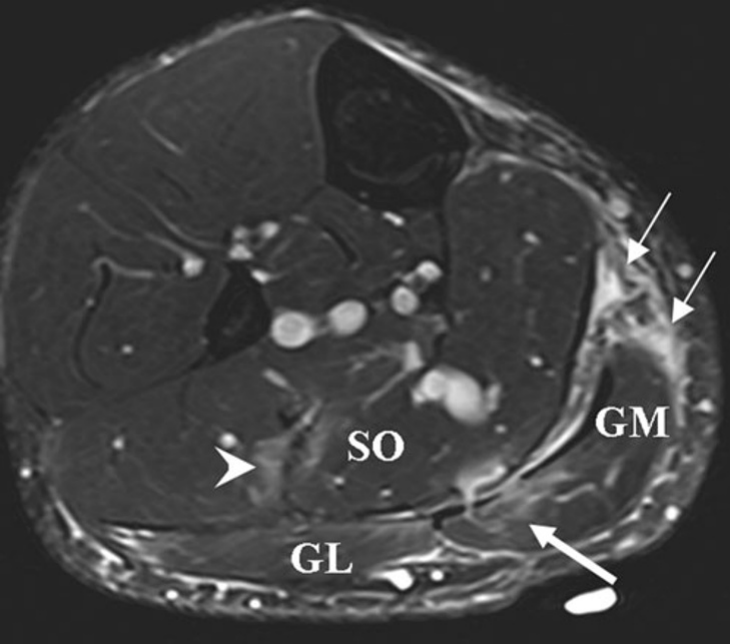
A 28-year-old tennis player presenting with calf pain, clinically diagnosed as a grade 1 injury. Axial FS T2-w TSE images demonstrate areas of hyperintensity with feathery appearance, consistent with a strain of the soleus muscle (*SO*, *arrowhead*) and also a strain of the medial head of the gastrocnemius muscle with intramuscular oedema (*GM*, *thick arrow*). Further epifascial haematoma and oedema at the posterior aspects of the medial gastrocnemius are depicted (*thin arrows*)

**Fig 3:**
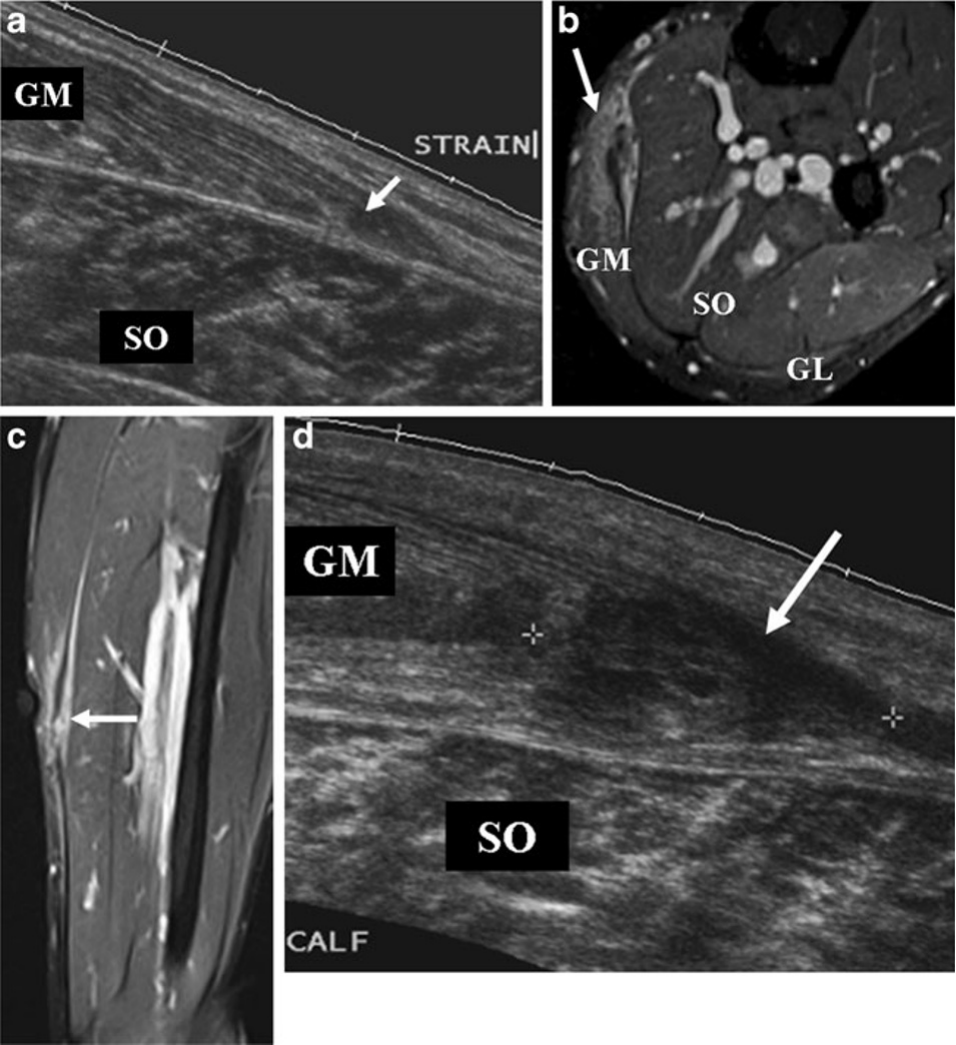
A 27-year-old football player presenting with left calf pain, clinically diagnosed as grade 2 injury. **a** On ultrasound at initial presentation, a hypoechoic area measuring 1.0 × 0.4 cm was noted (*arrow*), corresponding with a partial tear of the medial head of the gastrocnemius (*GM*). Colour Doppler imaging was normal (not shown). No evidence of haematoma or other abnormality was observed. Soleus muscle (*SO*) appears intact. MR imaging was not performed on this patient at this time. **b-d** Follow-up imaging 2 months after initial presentation. **b** Axial and (**c**) coronal FS T2-w TSE images reveal areas of hyperintensity with feathery appearance, consistent with a partial tear of the medial head of the gastrocnemius muscle with intramuscular oedema (*arrow*). Note the peritendinous oedema. **d** On ultrasound, a persistent hypoechogenic area was noted that appeared to have increased in extent (*arrow*). Colour Doppler imaging was normal (not shown). A 7-month follow-up MR imaging showed complete recovery (not shown)

**Fig 4:**
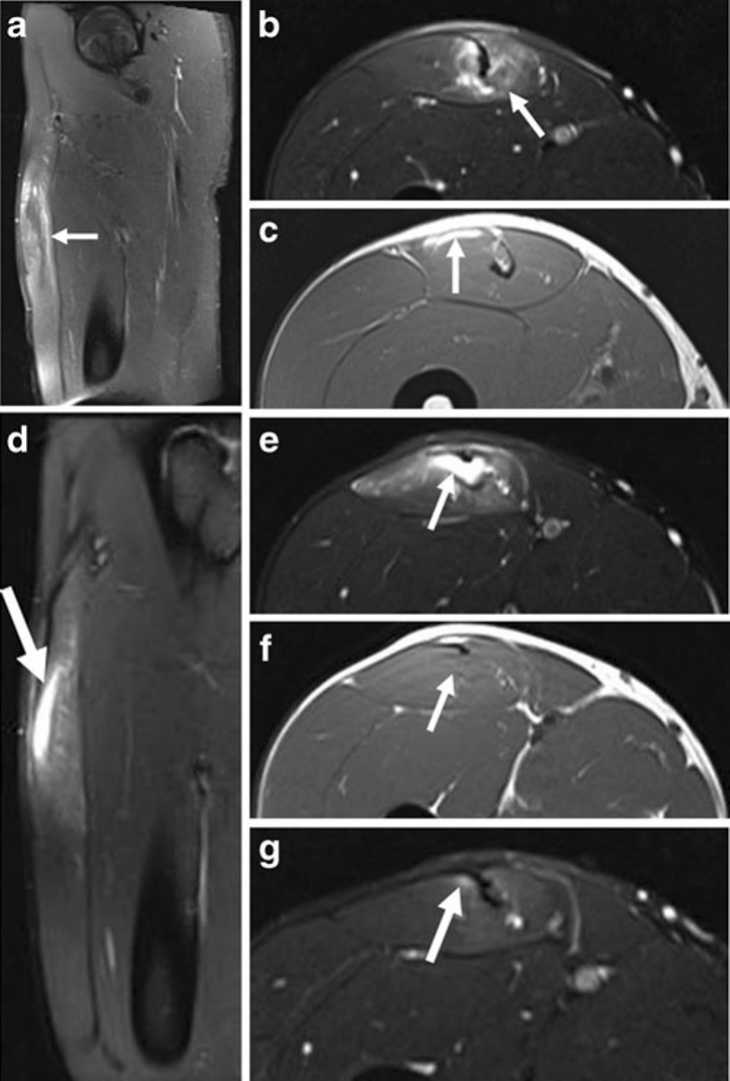
Follow-up MR images of a 20-year-old professional football player with a history of partial tear of the proximal third of the musculotendinous junction of the rectus femoris muscle 3 weeks prior. The patient presented with a re-injury after resuming competitive activity. **a** Sagittal and (**b**) axial FS T2-w images and (**c**) axial T1-w image. Extending distally from the proximal musculotendinous junction, a multisegmental intratendinous partial tear of the rectus femoris is demonstrated with significant peritendinous muscle oedema (*arrows*). **c** Epifascial intramuscular hyperintensity on T1-w image in the anterior rectus femoris represents acute haemorrhage (*arrow*). At 5-month follow-up, (**d**) sagittal and (**e**) axial FS T2-w images show focal, full-thickness tendon disruption involving the deep tendon of the rectus femoris muscle (*arrows*). There is a musculotendinous gap, filled with fluid (*arrow*), at the level of the mid-thigh measuring 3.4 cm in length. The fluid collection represents liquefied residual haematoma with hyperintensity on the (**e**, *arrow*) FS T2-w TSE image and isointensity to hypointensity on the (**f**, *arrow*) T1-w image. Some of the fibres of the intramuscular tendon remain intact. **g** Axial FS T2-w image at 6.5-month follow-up demonstrates only very discrete peritendinous oedema adjacent to the tendon (*arrow*)

Normal skeletal muscles show intermediate to low signal intensity on both T1-weighted (T1-w)(short TR/TE) and T2-weighted or short tau inversion recovery (STIR) (long TR/long TE) images compared with other soft tissues [28]. Alterations in water content in the affected musculotendinous units are common to all forms of acute traumatic injuries (Figs. 2, 3 and 4) [1–3]. Fluid-sensitive sequences, i.e., fat-suppressed T2-weighted (FS T2-w) or proton density-weighted (FS PD-w) turbo spin echo (TSE), and STIR sequences are suitable for detecting oedematous changes (hyperintensity with a ‘feathery’ appearance) in the musculotendinous unit, and to delineate and locate intramuscular or perifascial fluid collections or haematomas as hyperintensity [2, 29]. Such sequences can depict abnormal hyperintensities at the site of symptomatic old tears [30]. T1-w TSE sequences are used to visualise atrophy and fatty infiltration and to differentiate between haemorrhage/haematoma (hyperintensity) and oedema (hypointensity) [3], but they are less sensitive for depiction of soft tissue abnormalities (Fig. 4) [28]. In chronic muscle injuries, T1-w images may not show any signal abnormalities in small tears [30].

#### Ultrasound

**Fig 5:**
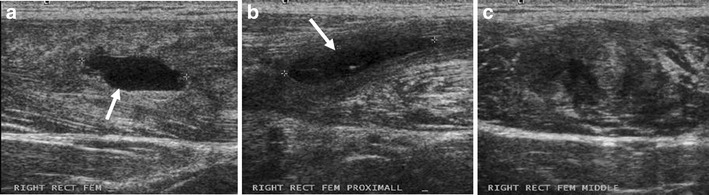
Ultrasound images of a 20-year-old football player (the same patient as the one shown in Fig. 4) taken at the same time as Fig. 4a–c. **a** A newly developed haematoma (*arrow*) measuring 2 cm × 0.8 cm is noted in the proximal mid-portion of the rectus femoris muscle. **b** More proximally, a known pre-existing fluid collection (2.5 cm × 0.5 cm, *arrow*) from the previous injury 3 weeks prior is observed. It is difficult to differentiate (**a**) as a new and (**b**) as an older lesion on the basis of ultrasound alone. **c** In between these two lesions, a notable echo inhomogeneity is noted, suggestive of a partial tear of the proximal rectus femoris muscle

Ultrasound is inexpensive and widely available, and some clinicians may prefer it to MR imaging for the initial assessment of injury in the clinic. Unlike MR imaging, ultrasound allows dynamic imaging while manoeuvring the injured leg to elicit symptoms and aid in clarifying the diagnosis. Power Doppler is useful for identifying hyperaemia associated with acute injuries [30]. Moreover, large haematoma may be drained under ultrasound guidance after liquefaction of the haematoma has occurred. The sensitivity of ultrasound to post-traumatic fluid collection in the acute stage has been shown to be equal to MR imaging [31]. However, the sensitivity for detecting ongoing muscle healing during recovery is not as high as MR [8]. A study involving Australian football players showed that follow-up MR imaging 6 weeks after hamstring injury detected persistent abnormalities in 36 % of athletes, whereas the 6-week follow-up ultrasound demonstrated residual abnormalities in only 24 % of patients. It is postulated that the lower sensitivity of ultrasound in prediction of convalescence time is due to underestimation of the degree of injury and cannot identify areas of subtle oedema. Overall, the disadvantages of ultrasound seem to outweigh the advantages compared with MR imaging especially for follow-up imaging [13], because it cannot differentiate between old and new lesions (Fig. 5) and is very difficult to reproduce exactly the same imaging position/plane at baseline and follow-up visits.

## Types of musculotendinous injuries and their imaging features

### Musculotendinous strains and tears

Musculotendinous strains and tears may be caused by a single traumatic event from excessive stretching on musculotendinous fiber (e.g., in high-speed runners) [17], from movements involving excessive range over sequential joints (e.g., in dancers) [32], or as a result of eccentric contractions (e.g., in football players) [33]. In some elite sports, such as track and field, football and rugby, the micro-damage from mild eccentric exercise may progress to more major tears [33]. The lesion is located at the musculotendinous junction, commonly in the superficial muscle layers, but the location may vary depending on the mechanism of injury. Musculotendinous strains can be clinically classified as grade 1, grade 2, and grade 3 based on absent, mild, or complete loss of muscle function, respectively [3]. These injury grades can be used to estimate the convalescent period and to design an appropriate rehabilitation program [34]. Muscle strains typically affect the muscles that extend across two joints, have a high proportion of fast-contracting type II fibres and fusiform shape, and undergo eccentric contractions [3, 35]. In the lower extremities, the hamstrings, rectus femoris and gastrocnemius muscles are commonly involved.

**Fig 6:**
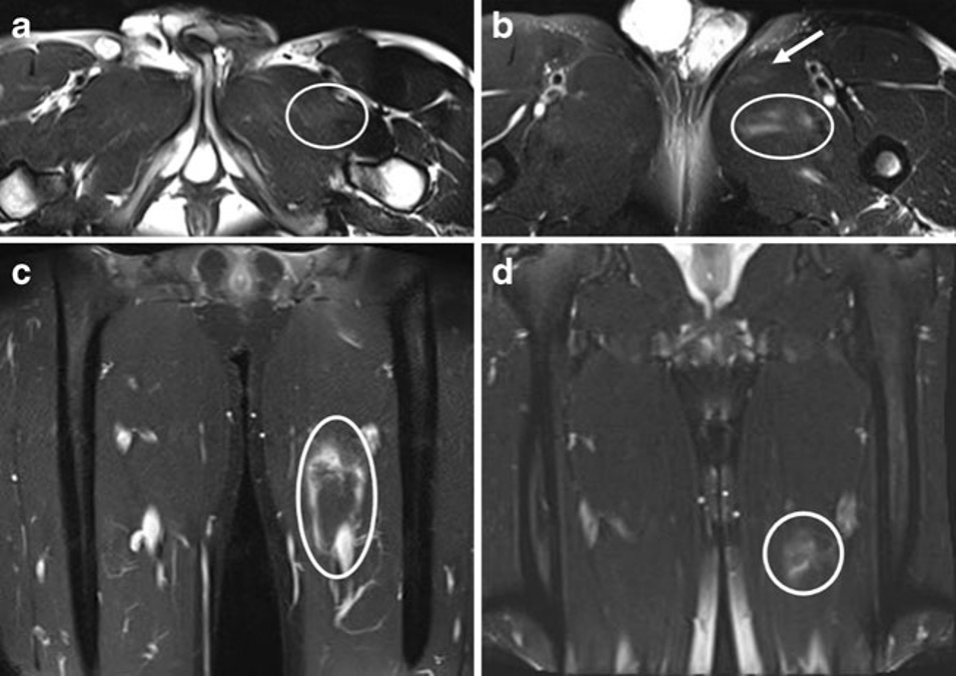
A 19-year-old gymnast presenting with an injury of the left groin. Axial FS T2-w TSE image shows (**a**) abnormal hyperintensity (*oval*) consistent with a strain of pectineus muscle, (**b**) discrete hyperintensity within the adductor longus (*arrow*, *strain without a tear*) and mild haematoma within the adductor brevis (*oval*, partial tear). (**c**) Haemorrhagic fluid extending into the fascia surrounding the adductor longus is shown (*oval*). The area of perifascial fluid spans a length of 10.8 cm over the proximal to mid aspects of the thigh. (**d**) The MR imaging at the 1-month follow-up showed some residual hyperintensity in the medial thigh (*circle*) corresponding with persistent oedema surrounding the adductor longus, but near-complete resolution of haematoma is noted. Normal homogeneous signal intensity was seen within the pectineus, adductor longus and brevis muscles, indicating recovery (not shown)

In musculotendinous strain without a tear, some fibre disruptions are seen as a result of a stretch injury, but muscle functions are maintained and treatment is conservative. On MR images, interstitial oedema and haemorrhage are present at the musculotendinous junction and extend into the adjacent muscle fascicles, producing a feathery appearance (i.e., hyperintensity) on fluid-sensitive sequences [28, 36] (Figs. 1, 2 and 6a). However, up to 45 % of clinically diagnosed grade 1 hamstring injuries may have a normal appearance on MR imaging according to one study [8]. On ultrasound, the lesion may be depicted as hyperechogenicity [37], or hypoechogenicity, or may appear normal [37].

In the presence of partial tears of fibres without retraction, there is a mild loss of muscle function. On MR images, in addition to interstitial oedema and haemorrhage, haematoma at the musculotendinous junction and perifascial fluid collection appear as hyperintensity on fluid-sensitive sequences (Figs. 3, 4 and 6b). On ultrasound, these pathologic features are depicted as hypoechogenicity (Figs. 3 and 5). Disruption of muscle fibres will be depicted as notable echo inhomogeneity (Fig. 8). Treatment of partial tears is also conservative.

**Fig 7:**
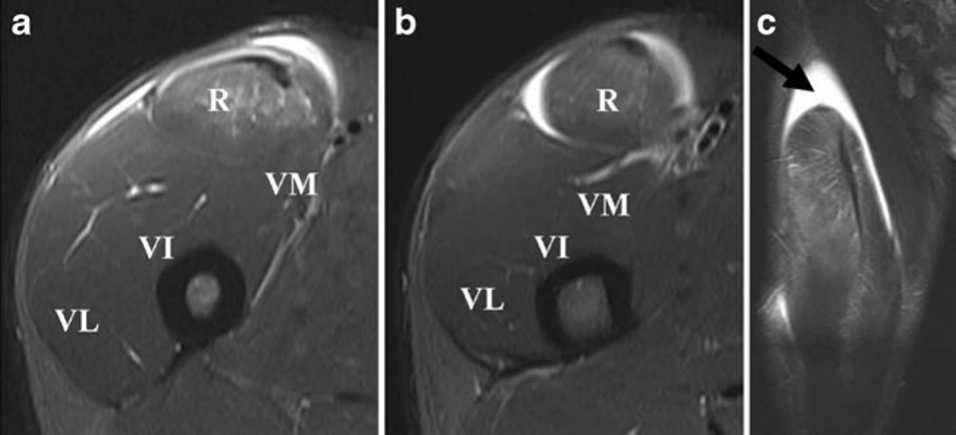
An 18-year-old rugby player (kicker) presenting with an acute right thigh injury after repeated kicking of the ball. **a**, **b** Axial and (**c**) coronal STIR images demonstrate a complete tear of the reflected head of the rectus femoris with surrounding haematoma. The coronal image demonstrates discontinuation of the reflected head of the rectus femoris at the proximal musculotendinous junction (*arrow*)

Complete musculotendinous rupture is commonly accompanied by a haematoma (Fig. 7). The diagnosis is usually made on clinical grounds, i.e., complete loss of muscle function, with palpable gap and muscle fiber retraction. Surgical repair is an option, depending on the location of the rupture [38], and both MR imaging and ultrasound may be useful for preoperative assessment of the extent of retraction [3]. Extensive acute oedema and haemorrhage may limit accurate evaluation of the injured muscle. If the tears are left untreated, the ends may become rounded and tether to adjacent muscles or fascia [39].

### Muscle contusion

Muscle contusions result from direct trauma, usually by a blunt object [35]. The injury consists of a well-defined sequence of events involving microscopic rupture and damage to muscle cells, macroscopic defects in muscle bellies, infiltrative bleeding, and inflammation. As a complication, myositis ossificans traumatica may develop [40]. Unlike strains, these traumas usually occur deep in the muscle belly and tend to be less symptomatic than strains. Severity depends on the site of impact, the activation status of the muscles involved, the age of the patient, and the presence of fatigue [41].

**Fig 8:**
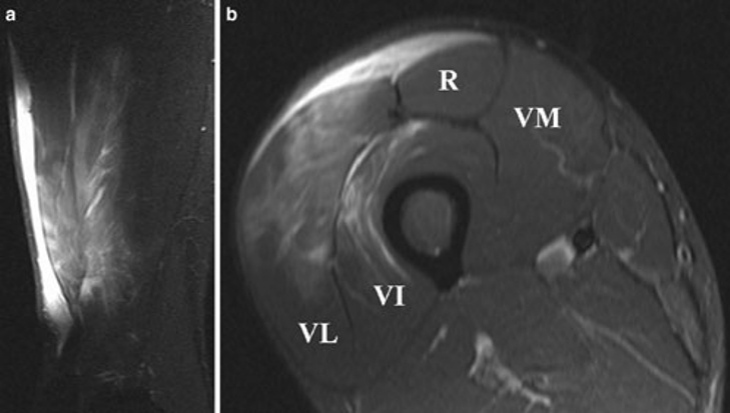
A 22-year-old rugby player presenting with stiffness of the right thigh after receiving a direct blow to the right anterior thigh during a tackle. **a** Sagittal and (**b**) axial STIR images show diffuse intramuscular hyperintensity, consistent with contusion injury of the rectus femoris, vastus lateralis and vastus intermedius muscles. Epifascial haematoma is noted superficially. The sagittal image demonstrates the longitudinal extent of the contusion injury and the haematoma

**Fig 9:**
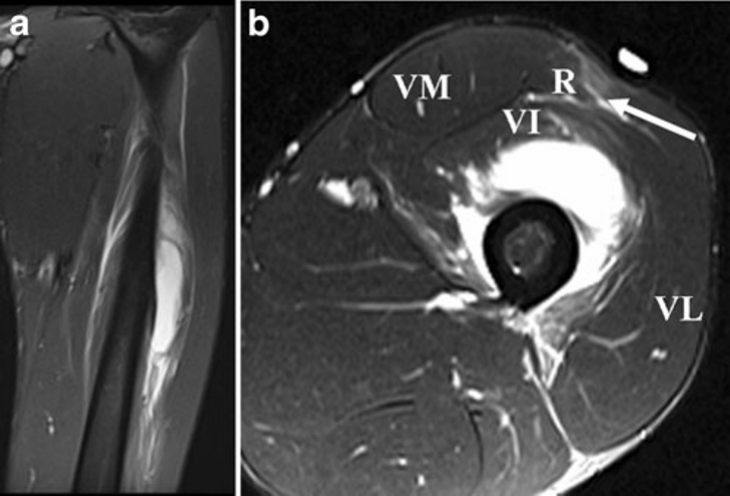
A 21-year-old football player presenting 5 days after a direct contusion injury to the left anterior thigh. **a** Coronal fat-suppressed (FS) T2-w TSE image reveal severe contusion injury of the vastus intermedius muscle, which extends to the proximal part of the musculotendinous junction of the quadriceps. The lesion is located at the middle part of the muscle and measures about 11 cm in long axis. The fluid-equivalent portion of the lesion represents a deep intramuscular haematoma. **b** Axial FS T2-w TSE image shows an additional strain of the rectus femoris muscle without a tear, depicted as hyperintensity surrounding the central musculotendinous junction at the mid-portion of the muscle (*arrow*)

On ultrasound, contusion is characterised by discontinuity of normal muscle architecture, with ill-defined hyperechogenicity that may cross fascial boundaries [37]. MR imaging varies according to severity of injury, but typically there is a feathery appearance of diffuse muscle oedema on STIR and FS T2-w images [1] (Fig. 8). Increased muscle girth can be observed but there are no other architectural changes, such as fibre discontinuity or laxity. In case of severe trauma with muscle fiber disruptions, deep intramuscular haematoma is seen [3] (Fig. 9). Signal intensity within the haematoma is influenced by the concentration of protein, methaemoglobin, magnetic susceptibility at high field strength, and tissue clearance [42]. Acute haematomas (<48 h) are typically isointense on T1-w images, and subacute haematomas (<30 days) appear hyperintense relative to muscle on both T1-w and fluid-sensitive sequences secondary to methaemoglobin accumulation [28]. As the haematoma evolves, a wide range of MR signal intensity can be seen within the collection, depending on the age of degradation products. Chronic haematoma characteristically shows a rim of hypointensity on all pulse sequences due to haemosiderin. As blood degradation products get reabsorbed over a course of 6–8 weeks, the size of haematoma will decrease [30].

### Avulsion injury

**Fig 10:**
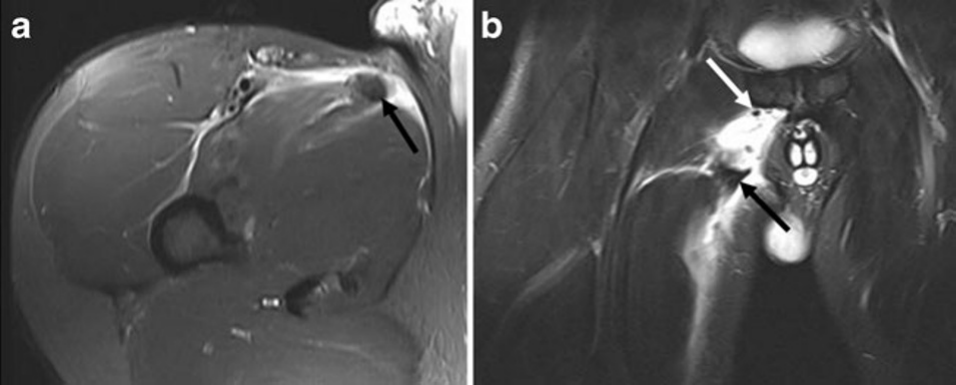
A 21-year-old male javelin thrower presenting with sudden onset of right-sided groin pain. **a** Axial and (**b**) coronal FS T2-w TSE images demonstrate a wavy appearance and retraction of the torn end of the adductor longus tendon (*black arrow*) with surrounding hyperintensity representing haematoma. Small hypointense fragments of avulsed cortical bone from the symphyseal attachment are noted in the coronal image (*white arrow*)

Acute avulsion injuries result from extreme, unbalanced and often eccentric muscular contractions, and patients with such injuries present with severe pain and loss of function [43]. Adolescents are particularly vulnerable to avulsion injuries because of the inherent weakness of the apophyses. The many apophyses in the pelvis and hip are common sites of avulsion injuries (Fig. 10). The single most common site of apophyseal avulsion is at the ischial tuberosity [44]. Cheerleaders, sprinters, gymnasts, track athletes, American football players, and baseball players are commonly affected [44]. Treatment for avulsion injury is generally conservative and the prognosis is good, but non-union may occur.

In acute avulsion injury, periosteal stripping with haematoma at a tendon attachment site can be depicted by MR imaging. A wavy appearance and retraction of the torn end of the tendon with fragments of bone/cartilage is characteristic. The redundant tendon edge may be lying in a large fluid collection/haematoma. Ultrasound evaluation is also useful, but may be difficult due to the presence of a mixed echogenicity haematoma which has echogenicity similar to the avulsed tendon [37].

### Chronic and repetitive injuries

Imaging features of chronic musculotendinous injury include muscle or tendon retraction or compensatory hypertrophy, muscle atrophy and formation of scar tissue (fibrosis) [45]. In chronic injuries, T1-w images may be normal in low-grade injuries, but the fluid-sensitive sequences are helpful for detection of symptomatic old tears which are depicted as abnormal hyperintensity [30]. There may be associated surrounding oedema and haemorrhage due to re-injury at the site (Fig. 4) [30]. Scar tissue may be observed as early as 6 weeks after initial injury [13]. On MR imaging, it appears as hypointensity on all pulse sequences and, on ultrasound, areas of scar tissue have irregular morphological features and show heterogeneous echogenicity [31]. It is important to identify the scar tissue because recurrent injuries can occur in the close proximity due likely to elasticity differences and altered contractility [30]. Imaging features of all the types of injuries discussed above are summarised in Table 1.

**Table 1:** Differentiation between new, old and recurrent injuries by imaging

Type of injury	Imaging findings	
	MR imaging	Ultrasound
New (acute)		
Grade 1 strain	• Intramuscular ‘feathery’ hyperintensity on fluid-sensitive sequences without muscle fibre disruption	○ Areas of intramuscular hyperechogenicity and perifascial hypoechogenicity (fluid collection)
Grade 2 strain	• Hyperintensity (oedema and haemorrhage) intramuscularly or at the MTJ, with extension along the fascial planes between muscle groups	○ Discontinuity of muscle fibres with hypervascularity around disrupted muscle fibres
	• Irregularity and mild laxity of tendon fibres	○ Altered echogenicity and loss of perimysial striation adjacent to the MTJ
	• Haematoma at the MTJ is pathognomonic	○ Intramuscular fluid collection (hypoechogenicity) with a surrounding hyperechoic halo
		○ Complete discontinuity of muscle fibers associated with extensive oedema and haematoma, and possible retraction of tendon
Grade 3 strain	• Complete discontinuity of muscle fibres associated with extensive oedema and haematoma, and possible retraction of tendon	○ Ill-defined area of hyperechogenicity in the muscle, which may cross fascial planes
Contusion	• T1-weighted and fluid-sensitive sequences may show hypo- to hyperintensity	
Hematoma	• Acute (<48 h): typically isointense to muscles on T1-weighted images	○ Appears as a hypoechoic fluid collection and may contain debris
	• Subacute (<30 days): higher signal intensity than muscle on both T1-weighted and fluid-sensitive sequences; variable signal intensities within hematoma	○ Variable appearance (anechoic, hypoechoic or hyperechoic) within 24 h of injury; appearance changes over the next few days becoming hypoechoic or anechoic
Avulsion	• Redundant tendon edge lying within large fluid collection/haematoma	○ Evaluation is difficult due to the presence of mixed echogenicity haematoma with similar echogenicity to the avulsed tendon
	• A small bony fragment	
Old (chronic)		
Muscle enlargement or atrophy	• Chronic avulsion has no surrounding fluid and tendon edges may be difficult to define	
Scar tissue	• Scar tissue appears hypointense on all pulse sequences	○ Areas of scar tissue have irregular morphological features and display heterogeneous echo texture
Chronic hematoma	• Dark signal intensity rim seen on all pulse sequences due to hemosiderin (chronic haematoma)	

*MTJ* musculotendinous junction

### Healing process of injured muscles

At follow-up, grade I injury may be manifest as a region of hyperechogenicity in up to 50 % of cases on ultrasound [46]. In such cases, normal healing is typically evident as a decrease in size or resolution of the area of hypoechogenicity, together with return of normal muscle architecture and echotexture [29]. More severe injuries may be characterised by the presence of hypoechoic regions indicative of fluid adjacent to muscle fibrils or the epimysium. Resolution or notable reduction in the amount of fluid is expected during the normal healing process. Any haematoma or fluid collection should decrease in size, and macroscopic muscle tears may show echogenicity of the margins of the tear as healing occurs. As the healing process progresses, small tears may fill with echogenic material, which is presumed to be scar tissue [29]. Scar tissue formation at the site of injury can be seen at 6 weeks [30]. However, ultrasound is less sensitive than MR imaging to residual muscle injury during follow-up [13].

MR imaging of healing muscle injuries typically show gradual resolution of fluid between muscle fascicles and in relation to the epimysium, together with gradual reduction in the extent and intensity of T2 signal within muscle. The degree of resolution of T2 hyperintensity resolution is variable depending on the severity of the initial injury, but in many cases MR signal abnormality has not resolved by 6 weeks despite return to competition, especially when the initial injury was severe. Persistent high T2 signal suggests ongoing healing and resolution of injury in keeping with the fact that the ultrastructural healing process continues for weeks to months, even after the time when athletes usually return to competition [29]. Hypointensity may also be seen during muscle healing, and reflects the formation of scar tissue and/or haemosiderin deposition following haemorrhage. These changes may contribute to the susceptibility artifacts seen on T2-w images during follow-up [30]. During the first few weeks of healing, there may be thickening and T2 hyperintensity of the central intramuscular tendon at the site of injury. As maturation of the scar occurs, T2 hypointensity replaces the hyperintensity. A recent study of athletes with grade I and II hamstring injuries, follow-up MRI was performed 5–23 months post-injury [45]. Hypointensity representing scar tissue was seen along the musculotendinous junction in 79 % (11/14) of subjects. Muscle volume reduction following the injury of long head of biceps was observed, but fatty infiltration was infrequently seen on these follow-up MR imaging [45].

### Advanced imaging techniques

Research efforts are ongoing to develop MR imaging techniques for assessment of skeletal muscle ultrastructure. Diffusion tensor imaging (DTI) can provide detailed information on muscle structural changes [5]. A recent study showed that the water diffusivity parameters of the thigh muscles were not influenced by the age or gender of healthy subjects. However, the hamstrings and the quadriceps did show differences in such parameters, which, the authors speculate, may reflect differences in hydration and muscular architecture [47]. Muscle fibre tracking with DTI has been used successfully to assess muscle damage [48] and to measure pennation angle [49]. Muscle is fairly uniform in bulk directionality and exhibits orderly arrangements on DTI. Following muscle injury, disturbance of the normal arrangement will be observed [5]. DTI and fiber tracking packages are sold by all the major MR imaging vendors and can be performed using routinely available clinical scanners. Thus, DTI may become a useful tool in our clinical practice in the future.

Additionally, the use of MR spectroscopy (MRS) using phosphorus [50] or sodium [51] to assess skeletal muscle disorders has been explored. Phosphorus (^31^P) MRS measures oxidative metabolism and sodium (^23^Na) MRS measures tissue sodium levels in muscle during exercise and recovery. These techniques may be useful as a noninvasive tool in exercise physiology or sports medicine to better understand the dynamics of an exercising muscle [5], but their applicability and relevance to evaluation of muscle injury are yet to be determined.

### Relevance of imaging

There are high demands on sports medicine physicians to quantify the prognosis and to predict when the athlete can return to training and competition. Imaging may assist in the prognostication of the healing process and in predicting the risk of recurrence, but the decision on return to play cannot be dependent on the imaging findings alone and must be balanced against the clinical situation [15, 52]. It has been shown that athletes with a normal MR imaging study in the presence of clinically suspected muscle injury require a shorter convalescence interval (1–2 weeks) [8] and have a lower recurrence rate [29] than those with an abnormal MR study. However, when there is an abnormality on MR or ultrasound, there is no conclusive evidence that the extent of the abnormality can predict the risk of recurrent injury, whether the images are acquired shortly after the injury or just prior to returning to competition [29].

Imaging parameters used to estimate the extent of muscle injury include the percentage of the cross-sectional area of the affected muscle, the craniocaudal length of the muscle lesion adjacent to the musculotendinous junction, and the approximate volume of muscle injury [29]. These parameters are associated with the duration of absence from competition and thus may guide clinicians in managing muscle injuries [9, 13, 53, 54]. Studies have shown that complete tear of the hamstring, as well as hamstring injuries involving >50 % of muscle cross-sectional area, haemorrhage, fluid collections and distal myotendinous involvement, were associated with a longer recovery time [53, 54]. A study of sprinters demonstrated that time to return to pre-injury level following hamstring injury involving the proximal tendon was significantly longer (approximately 35 weeks) than when the proximal tendon was not involved (less than 15 weeks) [17]. In regard to the quadriceps, sprinters with acute injury involving the central tendon of the rectus femoris was shown to have a mean recovery time of 26.85 days, which was significantly longer than those who sustained injury to the peripheral tendon of the rectus femoris (9.17 days) and vasti muscles (4.42 days). Other studies also showed that the larger the size of the injury on MR imaging the greater the risk of recurrence [12, 55]. Follow-up imaging may thus provide additional information to support clinical progress through a rehabilitation program [45], although many athletes will return to activity before the MR imaging findings are resolved [11] (Figs. 7, 8 and 9).

High quality imaging is of great clinical relevance in planning and guiding athlete rehabilitation. It is well established that MR imaging is the best technique for monitoring the muscle healing process, although this is tempered by the need to let the clinical evaluation guide return-to-play decisions [13]. No official guidelines on the role of MR imaging or ultrasound in evaluating muscle injuries in high-level athletes have been published. An algorithm that integrates clinical and imaging information into an explicit management plan would be very useful and needs to be developed [15, 29].

## Conclusion

Currently, MR imaging is the imaging method of choice for the initial diagnosis and follow-up of acute musculotendinous injuries, including strain, contusion and avulsion injuries. MR imaging may allow clinicians to more accurately estimate the time required before an athlete can return to competition as well as the risk of recurrent injury, but the available evidence for its specific utility remains limited and controversial. Ultrasound may play a role as an adjunct imaging method, but it is less accurate than MR imaging for assessing the extent of the injury and it cannot differentiate between new and old injuries. It is of great clinical importance to use high quality imaging to plan and guide athlete rehabilitation, although the clinical evaluation itself must guide return-to-play decisions.
